# Supplementary material for: Investigating Farm Fragmentation as a Risk Factor for Bovine Tuberculosis in Cattle Herds: A Matched Case-Control Study from Northern Ireland
Source: Pathogens. 2022 Feb 26;11(3):299. doi: 10.3390/pathogens11030299 (PMC8954255; doi:10.3390/pathogens11030299)
Supplement: Supplementary file 1 [file pathogens-11-00299-s001.zip › SM/SM_1.html]

DescAnalysisEpi


# DescAnalysisEpi

#### Georgina Milne

#### 01 Sep 2021

## R Markdown

This is an R Markdown document which should hopefully make the analytical steps clear and repeatable.

## 1. Descriptive Statistics

### TABLE 1

The following shows the distribution of explanatory variables between cases (bTBPOS) and controls (bTBNeg). The matching criteria where *Year*, *DVO*, *Median Herd Size* and *Herd Type*.

| **Variable** | **bTB Status** | |
| --- | --- | --- |
|  | **bTBNeg**, N = 4,6371 | **bTBPos**, N = 4,6371 |
| year |  |  |
| 2015 | 1,774 (38%) | 1,774 (38%) |
| 2016 | 1,389 (30%) | 1,389 (30%) |
| 2017 | 1,474 (32%) | 1,474 (32%) |
| DVO |  |  |
| Armagh | 428 (9.2%) | 428 (9.2%) |
| Ballymena | 269 (5.8%) | 269 (5.8%) |
| Coleraine | 590 (13%) | 590 (13%) |
| Dungannon | 590 (13%) | 590 (13%) |
| Enniskillen | 566 (12%) | 566 (12%) |
| Larne | 226 (4.9%) | 226 (4.9%) |
| Londonderry | 119 (2.6%) | 119 (2.6%) |
| Newry | 688 (15%) | 688 (15%) |
| Newtownards | 515 (11%) | 515 (11%) |
| Omagh | 646 (14%) | 646 (14%) |
| NewHerdType |  |  |
| Breeder | 1,251 (27%) | 1,251 (27%) |
| Dairy | 1,267 (27%) | 1,267 (27%) |
| Finisher | 690 (15%) | 690 (15%) |
| Other | 1,429 (31%) | 1,429 (31%) |
| median\_herd\_size | 81 (41, 156) | 83 (40, 173) |
| total\_farm\_area\_ha | 42 (26, 72) | 50 (27, 84) |
| farm\_area\_category |  |  |
| SmallArea | 583 (13%) | 538 (12%) |
| MediumArea | 938 (20%) | 852 (18%) |
| LargeArea | 1,570 (34%) | 1,343 (29%) |
| VeryLargeArea | 1,546 (33%) | 1,904 (41%) |
| n\_fields | 28 (17, 44) | 33 (20, 51) |
| n\_fragments\_5m | 4 (2, 6) | 4 (3, 7) |
| fragment\_category |  |  |
| Not\_fragmented | 526 (11%) | 488 (11%) |
| Little\_fragmentation | 2,226 (48%) | 1,974 (43%) |
| Medium\_fragmentation | 1,062 (23%) | 1,165 (25%) |
| High\_fragmentation | 415 (8.9%) | 551 (12%) |
| Very\_High\_fragmentation | 408 (8.8%) | 459 (9.9%) |
| median\_distance\_fragments\_km | 1.52 (0.72, 3.23) | 1.83 (0.86, 3.57) |
| fragment\_distance\_category |  |  |
| Low | 798 (17%) | 697 (15%) |
| Medium | 1,338 (29%) | 1,116 (24%) |
| High | 1,282 (28%) | 1,425 (31%) |
| Very High | 1,219 (26%) | 1,399 (30%) |
| total\_shared\_boundary\_grazing\_km | 3.4 (2.0, 5.7) | 4.1 (2.2, 6.7) |
| neighbour\_contact\_category |  |  |
| Low | 763 (16%) | 644 (14%) |
| Medium | 1,086 (23%) | 918 (20%) |
| High | 1,255 (27%) | 1,181 (25%) |
| Very High | 1,533 (33%) | 1,894 (41%) |
| NeighbourbTB | 2,463 (53%) | 3,284 (71%) |
| CountNeighbourbTB | 1 (0, 1) | 1 (0, 3) |
|  |  |  |
| --- | --- | --- |
| *1* n (%); Median (IQR) | | |

A plot of the distribution of same variables in the whole cattle population.

| **Variable** | **Population**, N = 57,0241 |
| --- | --- |
| year |  |
| 2015 | 19,008 (33%) |
| 2016 | 19,008 (33%) |
| 2017 | 19,008 (33%) |
| DVO |  |
| Armagh | 5,487 (9.6%) |
| Ballymena | 3,465 (6.1%) |
| Coleraine | 6,450 (11%) |
| Dungannon | 6,954 (12%) |
| Enniskillen | 7,848 (14%) |
| Larne | 3,882 (6.8%) |
| Londonderry | 2,061 (3.6%) |
| Newry | 8,934 (16%) |
| Newtownards | 4,458 (7.8%) |
| Omagh | 7,470 (13%) |
| Unknown | 15 |
| NewHerdType |  |
| Breeder | 28,415 (50%) |
| Dairy | 8,128 (14%) |
| Finisher | 5,976 (10%) |
| Other | 14,505 (25%) |
| median\_herd\_size | 40 (19, 89) |
| total\_farm\_area\_ha | 31 (16, 59) |
| farm\_area\_category |  |
| SmallArea | 14,164 (25%) |
| MediumArea | 14,353 (25%) |
| LargeArea | 14,255 (25%) |
| VeryLargeArea | 14,252 (25%) |
| n\_fields | 24 (14, 39) |
| n\_fragments\_5m | 3 (2, 6) |
| fragment\_category |  |
| High\_fragmentation | 4,691 (8.2%) |
| Little\_fragmentation | 27,164 (48%) |
| Medium\_fragmentation | 12,353 (22%) |
| Not\_fragmented | 9,476 (17%) |
| Very\_High\_fragmentation | 3,340 (5.9%) |
| median\_distance\_fragments\_km | 1.38 (0.52, 3.05) |
| fragment\_distance\_category |  |
| High | 14,255 (25%) |
| Low | 14,256 (25%) |
| Medium | 14,256 (25%) |
| Very High | 14,255 (25%) |
| Unknown | 2 |
| total\_shared\_boundary\_grazing\_km | 2,842 (1,477, 4,948) |
| neighbour\_contact\_category |  |
| High | 14,256 (25%) |
| Low | 14,256 (25%) |
| Medium | 14,256 (25%) |
| Very High | 14,256 (25%) |
| NeighbourbTB | 27,074 (48%) |
| Unknown | 510 |
|  |  |
| --- | --- |
| *1* n (%); Median (IQR) | |

SUPPLEMENTARY MATERIAL FIGURE 1

```
## OGR data source with driver: ESRI Shapefile 
## Source: "H:\Shapefiles\DVO_only", layer: "region_DVO"
## with 10 features
## It has 1 fields
```

## 2. Correlation between explanatory variables

Some of the potential explanatory variables are highly correlated, especially the *Contact Boundary* variable and *Number of Fragments*. The *Contact Boundary* is likely to be the most epidemiologically relevent but all will be investigated.

SUPPLEMENTARY MATERIAL FIGURE 2

## 3. UNIVARIABLE ASSOCIATIONS

## 3A FARM AREA

### 3A 1 Farm Area - Continuous

Exploratory loess plot of how mean bTB predicted positivity (binary outcome, 1/0) varies with the explanatory variable *Farm Area*. The relationship is not linear-there is a general increase in probability of bTB until the farm is around 500 ha in area, the a drop off. There is no biological reason for this which is not speculative.

Fig 3A in Manuscript

```
figMain3A
```

| **Characteristic** | **OR**1 | **95% CI**1 | **p-value** |
| --- | --- | --- | --- |
| total\_farm\_area\_ha | 1.00 | 1.00, 1.00 | 0.5 |
|  |  |  |  |
| --- | --- | --- | --- |
| *1* OR = Odds Ratio, CI = Confidence Interval | | | |


|  | loglik | Chisq | Df | Pr(>|Chi|) |
| --- | --- | --- | --- | --- |
| NULL | -3214.12 |  |  |  |
| total\_farm\_area\_ha | -3213.94 | 0.38 | 1 | 0.5397 |

How many farms biger than 500ha? Only 49

```
## [1] 49
```

### 3A 2 Farm Area - Categorical

| **Characteristic** | **OR**1 | **95% CI**1 | **p-value** |
| --- | --- | --- | --- |
| farm\_area\_category |  |  |  |
| SmallArea | — | — |  |
| MediumArea | 1.03 | 0.87, 1.21 | 0.8 |
| LargeArea | 1.09 | 0.92, 1.30 | 0.3 |
| VeryLargeArea | 1.87 | 1.55, 2.25 | <0.001 |
|  |  |  |  |
| --- | --- | --- | --- |
| *1* OR = Odds Ratio, CI = Confidence Interval | | | |

Only "Very Large Farms" are at elevated risk - dichotimise the variable at this point

| **Characteristic** | **OR**1 | **95% CI**1 | **p-value** |
| --- | --- | --- | --- |
| VeryLargeArea |  |  |  |
| SmallMediumLargeArea | — | — |  |
| VeryLargeArea | 1.74 | 1.56, 1.95 | <0.001 |
|  |  |  |  |
| --- | --- | --- | --- |
| *1* OR = Odds Ratio, CI = Confidence Interval | | | |

Is the farm area variable important in the model?


|  | loglik | Chisq | Df | Pr(>|Chi|) |
| --- | --- | --- | --- | --- |
| NULL | -3214.12 |  |  |  |
| farm\_area\_category | -3164.39 | 99.46 | 3 | 0.0000 |

Fig 3B in Manuscript

```
figMain3B
```

### 3A 3 Farm Area - Ordinal

For this, I create an ordered variable for farm size, and then compare a this (simpler) model to the more complex categorical model

| **Characteristic** | **OR**1 | **95% CI**1 | **p-value** |
| --- | --- | --- | --- |
| farm\_size\_category\_o | 1.27 | 1.19, 1.34 | <0.001 |
|  |  |  |  |
| --- | --- | --- | --- |
| *1* OR = Odds Ratio, CI = Confidence Interval | | | |

Is the ordinal variable better than the categorical one (if so could result in a simpler model) - Null hypothesis is that the simplest model is sufficient. P value is very low, which is evidence against the simpler (ordinal) model (-3182.8) and evidence for the more complex model with categorical predictors (-3164.4).


|  | loglik | Chisq | Df | P(>|Chi|) |
| --- | --- | --- | --- | --- |
| 1 | -3164.39 |  |  |  |
| 2 | -3182.76 | 36.74 | 2 | 0.0000 |

## 3B FARM FRAGMENTATION

### 3B 1 Farm Fragmentation - Continuous

Loess plot of how bTB predicted positively (binary outcome, 1/0) varies with the explanatory variable *Farm Fragmentation*. The relationship is not linear-there is a general increase in probability of bTB until arpund 10 fragments, a plauteau and then a sharp increase within the most fragmented farms.

Fig 3c in Manuscript

```
figMain3C
```

| **Characteristic** | **OR**1 | **95% CI**1 | **p-value** |
| --- | --- | --- | --- |
| n\_fragments\_5m | 1.03 | 1.02, 1.05 | <0.001 |
|  |  |  |  |
| --- | --- | --- | --- |
| *1* OR = Odds Ratio, CI = Confidence Interval | | | |


|  | loglik | Chisq | Df | Pr(>|Chi|) |
| --- | --- | --- | --- | --- |
| NULL | -3214.12 |  |  |  |
| n\_fragments\_5m | -3198.04 | 32.16 | 1 | 0.0000 |

### 3B 2 Farm Fragmentation - Categorical

| **Characteristic** | **OR**1 | **95% CI**1 | **p-value** |
| --- | --- | --- | --- |
| fragment\_category |  |  |  |
| Not\_fragmented | — | — |  |
| Little\_fragmentation | 0.97 | 0.84, 1.11 | 0.7 |
| Medium\_fragmentation | 1.25 | 1.07, 1.46 | 0.004 |
| High\_fragmentation | 1.56 | 1.30, 1.88 | <0.001 |
| Very\_High\_fragmentation | 1.36 | 1.12, 1.65 | 0.002 |
|  |  |  |  |
| --- | --- | --- | --- |
| *1* OR = Odds Ratio, CI = Confidence Interval | | | |

Is the farm fragmentation category variable important in the model?


|  | loglik | Chisq | Df | Pr(>|Chi|) |
| --- | --- | --- | --- | --- |
| NULL | -3214.12 |  |  |  |
| fragment\_category | -3187.45 | 53.34 | 4 | 0.0000 |

Fig 3D in Manuscript

```
figMain3D
```

### 3B 3 Farm Fragmentation - Ordinal

| **Characteristic** | **OR**1 | **95% CI**1 | **p-value** |
| --- | --- | --- | --- |
| fragment\_category\_o | 1.14 | 1.09, 1.19 | <0.001 |
|  |  |  |  |
| --- | --- | --- | --- |
| *1* OR = Odds Ratio, CI = Confidence Interval | | | |

Is the ordinal variable better than the categorical one (if so could result in a simpler model) - Null hypothesis is that the simplest model is sufficient. P value is very low, which is evidence against the simpler (ordinal) model (-3196) and evidence for the more complex model with categorical predictors (-3187.5).


|  | loglik | Chisq | Df | P(>|Chi|) |
| --- | --- | --- | --- | --- |
| 1 | -3187.45 |  |  |  |
| 2 | -3196.04 | 17.18 | 3 | 0.0006 |

## 3C FRAGMENT DISPESAL

### 3C 1 Fragment Dispersal - Continuous

Loess plot of how mean bTB predicted positivity (binary outcome, 1/0) varies with the explanatory variable *Fragment Dispersal*.

Fig 3E in Manuscript

```
figMain3E
```

Explanatory variable is scaled so that it represents the change per 10km

| **Characteristic** | **OR**1 | **95% CI**1 | **p-value** |
| --- | --- | --- | --- |
| median\_distance\_fragments\_km\_scaled | 1.00 | 1.00, 1.00 | 0.030 |
|  |  |  |  |
| --- | --- | --- | --- |
| *1* OR = Odds Ratio, CI = Confidence Interval | | | |


|  | loglik | Chisq | Df | Pr(>|Chi|) |
| --- | --- | --- | --- | --- |
| NULL | -3214.12 |  |  |  |
| median\_distance\_fragments\_km\_scaled | -3211.73 | 4.78 | 1 | 0.0287 |

### 3C 3 Fragment Dispersal - Categorical

### Conditional GLM

| **Characteristic** | **OR**1 | **95% CI**1 | **p-value** |
| --- | --- | --- | --- |
| fragment\_distance\_category |  |  |  |
| Low | — | — |  |
| Medium | 0.97 | 0.85, 1.11 | 0.6 |
| High | 1.33 | 1.16, 1.52 | <0.001 |
| Very High | 1.35 | 1.19, 1.55 | <0.001 |
|  |  |  |  |
| --- | --- | --- | --- |
| *1* OR = Odds Ratio, CI = Confidence Interval | | | |

Is the fragment distance category variable important in the model? Yes.


|  | loglik | Chisq | Df | Pr(>|Chi|) |
| --- | --- | --- | --- | --- |
| NULL | -3214.12 |  |  |  |
| fragment\_distance\_category | -3188.68 | 50.88 | 3 | 0.0000 |

Fig 3F in Manuscript

```
figMain3F
```

Farms with high or very high dispersal have similar risk - dichotimise the variable at this point

| **Characteristic** | **OR**1 | **95% CI**1 | **p-value** |
| --- | --- | --- | --- |
| HighVeryHighDisp |  |  |  |
| SmallMedium | — | — |  |
| HighVeryHigh | 1.37 | 1.25, 1.49 | <0.001 |
|  |  |  |  |
| --- | --- | --- | --- |
| *1* OR = Odds Ratio, CI = Confidence Interval | | | |

### 3C 3 Farm Fragmentation - Ordinal

| **Characteristic** | **OR**1 | **95% CI**1 | **p-value** |
| --- | --- | --- | --- |
| fragment\_distance\_category\_o | 1.14 | 1.09, 1.18 | <0.001 |
|  |  |  |  |
| --- | --- | --- | --- |
| *1* OR = Odds Ratio, CI = Confidence Interval | | | |

Is the (simpler) ordinal model sufficient? P value is very low, which is evidence against the simpler (ordinal) model (-3194.8) and evidence for the more complex model with categorical predictors (-3188.7).

  
  

|  | loglik | Chisq | Df | P(>|Chi|) |
| --- | --- | --- | --- | --- |
| 1 | -3188.68 |  |  |  |
| 2 | -3194.78 | 12.19 | 2 | 0.0023 |

## 3D CONTACT METRICS (i.e. shared boundary - exposure to neighbouring farms)

### 3D 1 Contact Metrics - Continuous

Loess plot of how bTB predicted positively (binary outcome, 1/0) varies with the explanatory variable *Contact Metrics*. The relationship is generally linear, with a a general decrease in probability of bTB as shared contact boundaries increases.

Fig 3G in Manuscript

```
figMain3G
```

| **Characteristic** | **OR**1 | **95% CI**1 | **p-value** |
| --- | --- | --- | --- |
| total\_shared\_boundary\_grazing\_km | 1.07 | 1.06, 1.09 | <0.001 |
|  |  |  |  |
| --- | --- | --- | --- |
| *1* OR = Odds Ratio, CI = Confidence Interval | | | |


|  | loglik | Chisq | Df | Pr(>|Chi|) |
| --- | --- | --- | --- | --- |
| NULL | -3214.12 |  |  |  |
| total\_shared\_boundary\_grazing\_km | -3164.68 | 98.88 | 1 | 0.0000 |

### 3D 3 Contact Metrics - Categorical

| **Characteristic** | **OR**1 | **95% CI**1 | **p-value** |
| --- | --- | --- | --- |
| neighbour\_contact\_category |  |  |  |
| Low | — | — |  |
| Medium | 1.02 | 0.89, 1.18 | 0.7 |
| High | 1.20 | 1.04, 1.38 | 0.011 |
| Very High | 1.72 | 1.49, 1.98 | <0.001 |
|  |  |  |  |
| --- | --- | --- | --- |
| *1* OR = Odds Ratio, CI = Confidence Interval | | | |

Is the contact category variable important in the model Yes.

Fig 3H in Manuscript

```
figMain3H
```


|  | loglik | Chisq | Df | Pr(>|Chi|) |
| --- | --- | --- | --- | --- |
| NULL | -3214.12 |  |  |  |
| neighbour\_contact\_category | -3169.94 | 88.36 | 3 | 0.0000 |

### 3D 4 Contact Metrics - Ordinal

| **Characteristic** | **OR**1 | **95% CI**1 | **p-value** |
| --- | --- | --- | --- |
| neighbour\_contact\_category\_o | 1.21 | 1.16, 1.27 | <0.001 |
|  |  |  |  |
| --- | --- | --- | --- |
| *1* OR = Odds Ratio, CI = Confidence Interval | | | |

Is the ordinal model sufficient? No. P value is very low, which is evidence against the simpler (ordinal) model (-3177.3) and evidence for the more complex model with categorical predictors (-3169.9).


|  | loglik | Chisq | Df | P(>|Chi|) |
| --- | --- | --- | --- | --- |
| 1 | -3169.94 |  |  |  |
| 2 | -3177.28 | 14.68 | 2 | 0.0006 |

## 3E NEIGHBOUR BTB STATUS

### 3E Continuous

Loess plot of how bTB predicted positively (binary outcome, 1/0) varies with the explanatory variable *number of bTB Positive neighbours*.

```
## Warning in simpleLoess(y, x, w, span, degree = degree, parametric =
## parametric, : pseudoinverse used at 0
```

```
## Warning in simpleLoess(y, x, w, span, degree = degree, parametric =
## parametric, : neighborhood radius 2
```

```
## Warning in simpleLoess(y, x, w, span, degree = degree, parametric =
## parametric, : reciprocal condition number 2.026e-015
```

```
## Warning in simpleLoess(y, x, w, span, degree = degree, parametric =
## parametric, : There are other near singularities as well. 1
```

Fig 3G in Manuscript

```
figMain3I
```

| **Characteristic** | **OR**1 | **95% CI**1 | **p-value** |
| --- | --- | --- | --- |
| CountNeighbourbTB | 1.45 | 1.40, 1.50 | <0.001 |
|  |  |  |  |
| --- | --- | --- | --- |
| *1* OR = Odds Ratio, CI = Confidence Interval | | | |


|  | loglik | Chisq | Df | Pr(>|Chi|) |
| --- | --- | --- | --- | --- |
| NULL | -3214.12 |  |  |  |
| CountNeighbourbTB | -2920.96 | 586.33 | 1 | 0.0000 |

How is the variable distributed between case and control herds?

```
## # A tibble: 2 x 3
##   bTBStatus   med    mx
##   <fct>     <int> <int>
## 1 bTBNeg        1    11
## 2 bTBPos        1    14
```

### 3E Binary

| **Characteristic** | **OR**1 | **95% CI**1 | **p-value** |
| --- | --- | --- | --- |
| NeighbourbTB | 2.20 | 2.01, 2.41 | <0.001 |
|  |  |  |  |
| --- | --- | --- | --- |
| *1* OR = Odds Ratio, CI = Confidence Interval | | | |

Is the count of bTB positive neighbours variable important in the model Yes.


|  | loglik | Chisq | Df | Pr(>|Chi|) |
| --- | --- | --- | --- | --- |
| NULL | -3214.12 |  |  |  |
| NeighbourbTB | -3056.18 | 315.88 | 1 | 0.0000 |

### 3D 4 Contact Metrics - Ordinal

| **Characteristic** | **OR**1 | **95% CI**1 | **p-value** |
| --- | --- | --- | --- |
| neighbour\_contact\_category\_o | 1.21 | 1.16, 1.27 | <0.001 |
|  |  |  |  |
| --- | --- | --- | --- |
| *1* OR = Odds Ratio, CI = Confidence Interval | | | |

Is the ordinal model sufficient? No. P value is very low, which is evidence against the simpler (ordinal) model (-3177.3) and evidence for the more complex model with categorical predictors (-3169.9).


|  | loglik | Chisq | Df | P(>|Chi|) |
| --- | --- | --- | --- | --- |
| 1 | -3169.94 |  |  |  |
| 2 | -3177.28 | 14.68 | 2 | 0.0006 |

## 4 Assess confounding & effect modification between each explanatory variable and bTB positive neighbours

### 4A FARM AREA

### 1. Continuous area & Binary neighbours (not in main MS)

| **Characteristic** | **OR**1 | **95% CI**1 | **p-value** |
| --- | --- | --- | --- |
| total\_farm\_area\_ha | 1.00 | 1.00, 1.00 | 0.2 |
| NeighbourbTB | 2.22 | 2.03, 2.43 | <0.001 |
|  |  |  |  |
| --- | --- | --- | --- |
| *1* OR = Odds Ratio, CI = Confidence Interval | | | |

| **Characteristic** | **OR**1 | **95% CI**1 | **p-value** |
| --- | --- | --- | --- |
| total\_farm\_area\_ha | 1.00 | 1.00, 1.00 | 0.002 |
| NeighbourbTB | 2.59 | 2.28, 2.93 | <0.001 |
| total\_farm\_area\_ha \* NeighbourbTB | 1.00 | 1.00, 1.00 | <0.001 |
|  |  |  |  |
| --- | --- | --- | --- |
| *1* OR = Odds Ratio, CI = Confidence Interval | | | |

| **Characteristic** | Unadjusted Analysis | | | Adjusted Analysis | | | Adjusted Analysis (Interaction | | |
| --- | --- | --- | --- | --- | --- | --- | --- | --- | --- |
|  | **OR**1 | **95% CI**1 | **p-value** | **OR**1 | **95% CI**1 | **p-value** | **OR**1 | **95% CI**1 | **p-value** |
| total\_farm\_area\_ha | 1.00 | 1.00, 1.00 | 0.5 | 1.00 | 1.00, 1.00 | 0.2 | 1.00 | 1.00, 1.00 | 0.002 |
| NeighbourbTB | 2.20 | 2.01, 2.41 | <0.001 | 2.22 | 2.03, 2.43 | <0.001 | 2.59 | 2.28, 2.93 | <0.001 |
| total\_farm\_area\_ha \* NeighbourbTB |  |  |  |  |  |  | 1.00 | 1.00, 1.00 | <0.001 |
|  |  |  |  |  |  |  |  |  |  |
| --- | --- | --- | --- | --- | --- | --- | --- | --- | --- |
| *1* OR = Odds Ratio, CI = Confidence Interval | | | | | | | | | |

### 2. Continuous area & Continuous neighbours (not in MS)

| **Characteristic** | **OR**1 | **95% CI**1 | **p-value** |
| --- | --- | --- | --- |
| total\_farm\_area\_ha | 1.00 | 1.00, 1.00 | 0.003 |
| CountNeighbourbTB | 1.46 | 1.41, 1.51 | <0.001 |
|  |  |  |  |
| --- | --- | --- | --- |
| *1* OR = Odds Ratio, CI = Confidence Interval | | | |

| **Characteristic** | **OR**1 | **95% CI**1 | **p-value** |
| --- | --- | --- | --- |
| total\_farm\_area\_ha | 1.00 | 1.00, 1.00 | 0.7 |
| CountNeighbourbTB | 1.51 | 1.44, 1.57 | <0.001 |
| total\_farm\_area\_ha \* CountNeighbourbTB | 1.00 | 1.00, 1.00 | 0.012 |
|  |  |  |  |
| --- | --- | --- | --- |
| *1* OR = Odds Ratio, CI = Confidence Interval | | | |

| **Characteristic** | Unadjusted Analysis | | | Adjusted Analysis | | | Adjusted Analysis (Interaction | | |
| --- | --- | --- | --- | --- | --- | --- | --- | --- | --- |
|  | **OR**1 | **95% CI**1 | **p-value** | **OR**1 | **95% CI**1 | **p-value** | **OR**1 | **95% CI**1 | **p-value** |
| total\_farm\_area\_ha | 1.00 | 1.00, 1.00 | 0.5 | 1.00 | 1.00, 1.00 | 0.003 | 1.00 | 1.00, 1.00 | 0.7 |
| CountNeighbourbTB | 1.45 | 1.40, 1.50 | <0.001 | 1.46 | 1.41, 1.51 | <0.001 | 1.51 | 1.44, 1.57 | <0.001 |
| total\_farm\_area\_ha \* CountNeighbourbTB |  |  |  |  |  |  | 1.00 | 1.00, 1.00 | 0.012 |
|  |  |  |  |  |  |  |  |  |  |
| --- | --- | --- | --- | --- | --- | --- | --- | --- | --- |
| *1* OR = Odds Ratio, CI = Confidence Interval | | | | | | | | | |

### 3. Categorical area & Binary neiehgbours (not in MS)

| **Characteristic** | **OR**1 | **95% CI**1 | **p-value** |
| --- | --- | --- | --- |
| farm\_area\_category |  |  |  |
| SmallArea | — | — |  |
| MediumArea | 0.88 | 0.74, 1.04 | 0.13 |
| LargeArea | 0.88 | 0.74, 1.06 | 0.2 |
| VeryLargeArea | 1.35 | 1.11, 1.65 | 0.003 |
| NeighbourbTB | 2.12 | 1.93, 2.33 | <0.001 |
|  |  |  |  |
| --- | --- | --- | --- |
| *1* OR = Odds Ratio, CI = Confidence Interval | | | |

| **Characteristic** | **OR**1 | **95% CI**1 | **p-value** |
| --- | --- | --- | --- |
| farm\_area\_category |  |  |  |
| SmallArea | — | — |  |
| MediumArea | 1.24 | 0.98, 1.55 | 0.069 |
| LargeArea | 1.03 | 0.82, 1.30 | 0.8 |
| VeryLargeArea | 1.80 | 1.40, 2.32 | <0.001 |
| NeighbourbTB | 3.65 | 2.78, 4.79 | <0.001 |
| farm\_area\_category \* NeighbourbTB |  |  |  |
| MediumArea \* NeighbourbTB | 0.45 | 0.32, 0.63 | <0.001 |
| LargeArea \* NeighbourbTB | 0.62 | 0.45, 0.85 | 0.003 |
| VeryLargeArea \* NeighbourbTB | 0.51 | 0.38, 0.71 | <0.001 |
|  |  |  |  |
| --- | --- | --- | --- |
| *1* OR = Odds Ratio, CI = Confidence Interval | | | |

| **Characteristic** | Unadjusted Analysis | | | Adjusted Analysis | | | Adjusted Analysis (Interaction | | |
| --- | --- | --- | --- | --- | --- | --- | --- | --- | --- |
|  | **OR**1 | **95% CI**1 | **p-value** | **OR**1 | **95% CI**1 | **p-value** | **OR**1 | **95% CI**1 | **p-value** |
| farm\_area\_category |  |  |  |  |  |  |  |  |  |
| SmallArea | — | — |  | — | — |  | — | — |  |
| MediumArea | 1.03 | 0.87, 1.21 | 0.8 | 0.88 | 0.74, 1.04 | 0.13 | 1.24 | 0.98, 1.55 | 0.069 |
| LargeArea | 1.09 | 0.92, 1.30 | 0.3 | 0.88 | 0.74, 1.06 | 0.2 | 1.03 | 0.82, 1.30 | 0.8 |
| VeryLargeArea | 1.87 | 1.55, 2.25 | <0.001 | 1.35 | 1.11, 1.65 | 0.003 | 1.80 | 1.40, 2.32 | <0.001 |
| NeighbourbTB | 2.20 | 2.01, 2.41 | <0.001 | 2.12 | 1.93, 2.33 | <0.001 | 3.65 | 2.78, 4.79 | <0.001 |
| farm\_area\_category \* NeighbourbTB |  |  |  |  |  |  |  |  |  |
| MediumArea \* NeighbourbTB |  |  |  |  |  |  | 0.45 | 0.32, 0.63 | <0.001 |
| LargeArea \* NeighbourbTB |  |  |  |  |  |  | 0.62 | 0.45, 0.85 | 0.003 |
| VeryLargeArea \* NeighbourbTB |  |  |  |  |  |  | 0.51 | 0.38, 0.71 | <0.001 |
|  |  |  |  |  |  |  |  |  |  |
| --- | --- | --- | --- | --- | --- | --- | --- | --- | --- |
| *1* OR = Odds Ratio, CI = Confidence Interval | | | | | | | | | |

### 4. Categorical area & Continuous neighbours (not in MS)

| **Characteristic** | **OR**1 | **95% CI**1 | **p-value** |
| --- | --- | --- | --- |
| farm\_area\_category |  |  |  |
| SmallArea | — | — |  |
| MediumArea | 0.90 | 0.76, 1.07 | 0.2 |
| LargeArea | 0.85 | 0.71, 1.02 | 0.085 |
| VeryLargeArea | 1.18 | 0.96, 1.44 | 0.11 |
| CountNeighbourbTB | 1.43 | 1.38, 1.48 | <0.001 |
|  |  |  |  |
| --- | --- | --- | --- |
| *1* OR = Odds Ratio, CI = Confidence Interval | | | |

| **Characteristic** | **OR**1 | **95% CI**1 | **p-value** |
| --- | --- | --- | --- |
| farm\_area\_category |  |  |  |
| SmallArea | — | — |  |
| MediumArea | 1.13 | 0.92, 1.39 | 0.2 |
| LargeArea | 0.93 | 0.75, 1.15 | 0.5 |
| VeryLargeArea | 1.43 | 1.14, 1.79 | 0.002 |
| CountNeighbourbTB | 1.90 | 1.61, 2.22 | <0.001 |
| farm\_area\_category \* CountNeighbourbTB |  |  |  |
| MediumArea \* CountNeighbourbTB | 0.69 | 0.57, 0.83 | <0.001 |
| LargeArea \* CountNeighbourbTB | 0.80 | 0.67, 0.95 | 0.010 |
| VeryLargeArea \* CountNeighbourbTB | 0.73 | 0.62, 0.87 | <0.001 |
|  |  |  |  |
| --- | --- | --- | --- |
| *1* OR = Odds Ratio, CI = Confidence Interval | | | |

| **Characteristic** | Unadjusted Analysis | | | Adjusted Analysis | | | Adjusted Analysis (Interaction | | |
| --- | --- | --- | --- | --- | --- | --- | --- | --- | --- |
|  | **OR**1 | **95% CI**1 | **p-value** | **OR**1 | **95% CI**1 | **p-value** | **OR**1 | **95% CI**1 | **p-value** |
| farm\_area\_category |  |  |  |  |  |  |  |  |  |
| SmallArea | — | — |  | — | — |  | — | — |  |
| MediumArea | 1.03 | 0.87, 1.21 | 0.8 | 0.90 | 0.76, 1.07 | 0.2 | 1.13 | 0.92, 1.39 | 0.2 |
| LargeArea | 1.09 | 0.92, 1.30 | 0.3 | 0.85 | 0.71, 1.02 | 0.085 | 0.93 | 0.75, 1.15 | 0.5 |
| VeryLargeArea | 1.87 | 1.55, 2.25 | <0.001 | 1.18 | 0.96, 1.44 | 0.11 | 1.43 | 1.14, 1.79 | 0.002 |
| CountNeighbourbTB | 1.45 | 1.40, 1.50 | <0.001 | 1.43 | 1.38, 1.48 | <0.001 | 1.90 | 1.61, 2.22 | <0.001 |
| farm\_area\_category \* CountNeighbourbTB |  |  |  |  |  |  |  |  |  |
| MediumArea \* CountNeighbourbTB |  |  |  |  |  |  | 0.69 | 0.57, 0.83 | <0.001 |
| LargeArea \* CountNeighbourbTB |  |  |  |  |  |  | 0.80 | 0.67, 0.95 | 0.010 |
| VeryLargeArea \* CountNeighbourbTB |  |  |  |  |  |  | 0.73 | 0.62, 0.87 | <0.001 |
|  |  |  |  |  |  |  |  |  |  |
| --- | --- | --- | --- | --- | --- | --- | --- | --- | --- |
| *1* OR = Odds Ratio, CI = Confidence Interval | | | | | | | | | |

### **5. Binary Area & Continuous neighbours (in MS)**

| **Characteristic** | **OR**1 | **95% CI**1 | **p-value** |
| --- | --- | --- | --- |
| VeryLargeArea |  |  |  |
| SmallMediumLargeArea | — | — |  |
| VeryLargeArea | 1.35 | 1.20, 1.52 | <0.001 |
| CountNeighbourbTB | 1.43 | 1.38, 1.48 | <0.001 |
|  |  |  |  |
| --- | --- | --- | --- |
| *1* OR = Odds Ratio, CI = Confidence Interval | | | |

| **Characteristic** | **OR**1 | **95% CI**1 | **p-value** |
| --- | --- | --- | --- |
| VeryLargeArea |  |  |  |
| SmallMediumLargeArea | — | — |  |
| VeryLargeArea | 1.46 | 1.25, 1.69 | <0.001 |
| CountNeighbourbTB | 1.47 | 1.40, 1.54 | <0.001 |
| VeryLargeArea \* CountNeighbourbTB |  |  |  |
| VeryLargeArea \* CountNeighbourbTB | 0.94 | 0.88, 1.01 | 0.10 |
|  |  |  |  |
| --- | --- | --- | --- |
| *1* OR = Odds Ratio, CI = Confidence Interval | | | |

The univariable model is improved by the addition of bTB positive neighbours


|  | loglik | Chisq | Df | P(>|Chi|) |
| --- | --- | --- | --- | --- |
| 1 | -3165.04 |  |  |  |
| 2 | -2908.74 | 512.60 | 1 | 0.0000 |

No evidence that an interaction term is needed


|  | loglik | Chisq | Df | P(>|Chi|) |
| --- | --- | --- | --- | --- |
| 1 | -2908.74 |  |  |  |
| 2 | -2907.38 | 2.73 | 1 | 0.0987 |

| **Characteristic** | Unadjusted Analysis | | | Adjusted Analysis | | | Adjusted Analysis (Interaction | | |
| --- | --- | --- | --- | --- | --- | --- | --- | --- | --- |
|  | **OR**1 | **95% CI**1 | **p-value** | **OR**1 | **95% CI**1 | **p-value** | **OR**1 | **95% CI**1 | **p-value** |
| VeryLargeArea |  |  |  |  |  |  |  |  |  |
| SmallMediumLargeArea | — | — |  | — | — |  | — | — |  |
| VeryLargeArea | 1.74 | 1.56, 1.95 | <0.001 | 1.35 | 1.20, 1.52 | <0.001 | 1.46 | 1.25, 1.69 | <0.001 |
| CountNeighbourbTB | 1.45 | 1.40, 1.50 | <0.001 | 1.43 | 1.38, 1.48 | <0.001 | 1.47 | 1.40, 1.54 | <0.001 |
| VeryLargeArea \* CountNeighbourbTB |  |  |  |  |  |  |  |  |  |
| VeryLargeArea \* CountNeighbourbTB |  |  |  |  |  |  | 0.94 | 0.88, 1.01 | 0.10 |
|  |  |  |  |  |  |  |  |  |  |
| --- | --- | --- | --- | --- | --- | --- | --- | --- | --- |
| *1* OR = Odds Ratio, CI = Confidence Interval | | | | | | | | | |

coefficient changes - 10% rule for farm area and neighbour bTB status

There is some confounding between area and neighbours with bTB - after controlling for bTB status of neighbours, the relationship between area and bTB risk is smaller

```
## [1] "22% difference between non adjusted and asjusted coefficient"
```

```
## [1] "1% difference between non adjusted and asjusted coefficient"
```

Explore confounding more - what's going on?

Larger farms more are likely to have bTB positive neighbours. The table below shows that 75% of larger farms have at least 1 bTB positive neighbour, compared to only 37% of smaller farms

```
## # A tibble: 4 x 5
##   farm_area_category    Q1   med    Q3   max
##   <fct>              <dbl> <dbl> <dbl> <dbl>
## 1 SmallArea              0     0     1     5
## 2 MediumArea             0     1     1     8
## 3 LargeArea              0     1     2    11
## 4 VeryLargeArea          1     1     3    14
```

```
Fig4Area
```

Looking only at the presence/absence of bTB positive neighbours - almost twice as many very large farms had at least one bTB positive neighbour.

| **Variable** | **SmallArea**, N = 1,1211 | **MediumArea**, N = 1,7901 | **LargeArea**, N = 2,9131 | **VeryLargeArea**, N = 3,4501 |
| --- | --- | --- | --- | --- |
| NeighbourbTB | 414 (37%) | 956 (53%) | 1,776 (61%) | 2,601 (75%) |
|  |  |  |  |  |
| --- | --- | --- | --- | --- |
| *1* n (%) | | | | |

### 4B FARM FRAGMENTATION

### 1. Continuous fragmentation & binary neighbours (not in MS)

| **Characteristic** | **OR**1 | **95% CI**1 | **p-value** |
| --- | --- | --- | --- |
| NeighbourbTB | 2.16 | 1.97, 2.37 | <0.001 |
| n\_fragments\_5m | 1.01 | 1.00, 1.03 | 0.020 |
|  |  |  |  |
| --- | --- | --- | --- |
| *1* OR = Odds Ratio, CI = Confidence Interval | | | |

| **Characteristic** | **OR**1 | **95% CI**1 | **p-value** |
| --- | --- | --- | --- |
| NeighbourbTB | 2.16 | 1.86, 2.51 | <0.001 |
| n\_fragments\_5m | 1.01 | 0.99, 1.04 | 0.3 |
| NeighbourbTB \* n\_fragments\_5m | 1.00 | 0.97, 1.03 | >0.9 |
|  |  |  |  |
| --- | --- | --- | --- |
| *1* OR = Odds Ratio, CI = Confidence Interval | | | |

| **Characteristic** | Unadjusted Analysis | | | Adjusted Analysis | | | Adjusted Analysis (Interaction | | |
| --- | --- | --- | --- | --- | --- | --- | --- | --- | --- |
|  | **OR**1 | **95% CI**1 | **p-value** | **OR**1 | **95% CI**1 | **p-value** | **OR**1 | **95% CI**1 | **p-value** |
| n\_fragments\_5m | 1.03 | 1.02, 1.05 | <0.001 | 1.01 | 1.00, 1.03 | 0.020 | 1.01 | 0.99, 1.04 | 0.3 |
| NeighbourbTB | 2.20 | 2.01, 2.41 | <0.001 | 2.16 | 1.97, 2.37 | <0.001 | 2.16 | 1.86, 2.51 | <0.001 |
| NeighbourbTB \* n\_fragments\_5m |  |  |  |  |  |  | 1.00 | 0.97, 1.03 | >0.9 |
|  |  |  |  |  |  |  |  |  |  |
| --- | --- | --- | --- | --- | --- | --- | --- | --- | --- |
| *1* OR = Odds Ratio, CI = Confidence Interval | | | | | | | | | |

### 2. Continuous fragmentation & Continuous neighbours (not in MS)

| **Characteristic** | **OR**1 | **95% CI**1 | **p-value** |
| --- | --- | --- | --- |
| CountNeighbourbTB | 1.45 | 1.40, 1.51 | <0.001 |
| n\_fragments\_5m | 0.99 | 0.98, 1.00 | 0.2 |
|  |  |  |  |
| --- | --- | --- | --- |
| *1* OR = Odds Ratio, CI = Confidence Interval | | | |

| **Characteristic** | **OR**1 | **95% CI**1 | **p-value** |
| --- | --- | --- | --- |
| CountNeighbourbTB | 1.51 | 1.44, 1.59 | <0.001 |
| n\_fragments\_5m | 1.00 | 0.99, 1.02 | 0.7 |
| CountNeighbourbTB \* n\_fragments\_5m | 0.99 | 0.99, 1.00 | 0.019 |
|  |  |  |  |
| --- | --- | --- | --- |
| *1* OR = Odds Ratio, CI = Confidence Interval | | | |

| **Characteristic** | Unadjusted Analysis | | | Adjusted Analysis | | | Adjusted Analysis (Interaction | | |
| --- | --- | --- | --- | --- | --- | --- | --- | --- | --- |
|  | **OR**1 | **95% CI**1 | **p-value** | **OR**1 | **95% CI**1 | **p-value** | **OR**1 | **95% CI**1 | **p-value** |
| n\_fragments\_5m | 1.03 | 1.02, 1.05 | <0.001 | 0.99 | 0.98, 1.00 | 0.2 | 1.00 | 0.99, 1.02 | 0.7 |
| CountNeighbourbTB | 1.45 | 1.40, 1.50 | <0.001 | 1.45 | 1.40, 1.51 | <0.001 | 1.51 | 1.44, 1.59 | <0.001 |
| CountNeighbourbTB \* n\_fragments\_5m |  |  |  |  |  |  | 0.99 | 0.99, 1.00 | 0.019 |
|  |  |  |  |  |  |  |  |  |  |
| --- | --- | --- | --- | --- | --- | --- | --- | --- | --- |
| *1* OR = Odds Ratio, CI = Confidence Interval | | | | | | | | | |

### 3. Categorical fragmentation and binary neighbours (not in MS)

| **Characteristic** | **OR**1 | **95% CI**1 | **p-value** |
| --- | --- | --- | --- |
| NeighbourbTB | 2.16 | 1.97, 2.37 | <0.001 |
| fragment\_category |  |  |  |
| Not\_fragmented | — | — |  |
| Little\_fragmentation | 0.88 | 0.77, 1.02 | 0.094 |
| Medium\_fragmentation | 1.05 | 0.90, 1.23 | 0.6 |
| High\_fragmentation | 1.28 | 1.05, 1.55 | 0.013 |
| Very\_High\_fragmentation | 1.01 | 0.83, 1.24 | 0.9 |
|  |  |  |  |
| --- | --- | --- | --- |
| *1* OR = Odds Ratio, CI = Confidence Interval | | | |

| **Characteristic** | **OR**1 | **95% CI**1 | **p-value** |
| --- | --- | --- | --- |
| NeighbourbTB | 1.68 | 1.30, 2.17 | <0.001 |
| fragment\_category |  |  |  |
| Not\_fragmented | — | — |  |
| Little\_fragmentation | 0.76 | 0.62, 0.93 | 0.007 |
| Medium\_fragmentation | 0.96 | 0.76, 1.22 | 0.7 |
| High\_fragmentation | 1.20 | 0.88, 1.65 | 0.3 |
| Very\_High\_fragmentation | 0.71 | 0.47, 1.08 | 0.11 |
| NeighbourbTB \* fragment\_category |  |  |  |
| NeighbourbTB \* Little\_fragmentation | 1.37 | 1.03, 1.83 | 0.030 |
| NeighbourbTB \* Medium\_fragmentation | 1.24 | 0.90, 1.71 | 0.2 |
| NeighbourbTB \* High\_fragmentation | 1.20 | 0.81, 1.78 | 0.4 |
| NeighbourbTB \* Very\_High\_fragmentation | 1.72 | 1.06, 2.77 | 0.027 |
|  |  |  |  |
| --- | --- | --- | --- |
| *1* OR = Odds Ratio, CI = Confidence Interval | | | |

The univariable model is improved by the addition of bTB positive neighbours


|  | loglik | Chisq | Df | P(>|Chi|) |
| --- | --- | --- | --- | --- |
| 1 | -3187.45 |  |  |  |
| 2 | -3043.59 | 287.72 | 1 | 0.0000 |

No evidence that an interaction term is needed

```
## <!-- html table generated in R 4.1.0 by xtable 1.8-4 package -->
## <!-- Tue Sep 14 11:50:35 2021 -->
## <table border=1>
## <tr> <th>  </th> <th> loglik </th> <th> Chisq </th> <th> Df </th> <th> P(&gt;|Chi|) </th>  </tr>
##   <tr> <td> 1 </td> <td align="right"> -3043.59 </td> <td align="right">  </td> <td align="right">  </td> <td align="right">  </td> </tr>
##   <tr> <td> 2 </td> <td align="right"> -3040.05 </td> <td align="right"> 7.08 </td> <td align="right"> 4 </td> <td align="right"> 0.1315 </td> </tr>
##    </table>
```

| **Characteristic** | Unadjusted Analysis | | | Adjusted Analysis | | | Adjusted Analysis (Interaction | | |
| --- | --- | --- | --- | --- | --- | --- | --- | --- | --- |
|  | **OR**1 | **95% CI**1 | **p-value** | **OR**1 | **95% CI**1 | **p-value** | **OR**1 | **95% CI**1 | **p-value** |
| fragment\_category |  |  |  |  |  |  |  |  |  |
| Not\_fragmented | — | — |  | — | — |  | — | — |  |
| Little\_fragmentation | 0.97 | 0.84, 1.11 | 0.7 | 0.88 | 0.77, 1.02 | 0.094 | 0.76 | 0.62, 0.93 | 0.007 |
| Medium\_fragmentation | 1.25 | 1.07, 1.46 | 0.004 | 1.05 | 0.90, 1.23 | 0.6 | 0.96 | 0.76, 1.22 | 0.7 |
| High\_fragmentation | 1.56 | 1.30, 1.88 | <0.001 | 1.28 | 1.05, 1.55 | 0.013 | 1.20 | 0.88, 1.65 | 0.3 |
| Very\_High\_fragmentation | 1.36 | 1.12, 1.65 | 0.002 | 1.01 | 0.83, 1.24 | 0.9 | 0.71 | 0.47, 1.08 | 0.11 |
| NeighbourbTB | 2.20 | 2.01, 2.41 | <0.001 | 2.16 | 1.97, 2.37 | <0.001 | 1.68 | 1.30, 2.17 | <0.001 |
| NeighbourbTB \* fragment\_category |  |  |  |  |  |  |  |  |  |
| NeighbourbTB \* Little\_fragmentation |  |  |  |  |  |  | 1.37 | 1.03, 1.83 | 0.030 |
| NeighbourbTB \* Medium\_fragmentation |  |  |  |  |  |  | 1.24 | 0.90, 1.71 | 0.2 |
| NeighbourbTB \* High\_fragmentation |  |  |  |  |  |  | 1.20 | 0.81, 1.78 | 0.4 |
| NeighbourbTB \* Very\_High\_fragmentation |  |  |  |  |  |  | 1.72 | 1.06, 2.77 | 0.027 |
|  |  |  |  |  |  |  |  |  |  |
| --- | --- | --- | --- | --- | --- | --- | --- | --- | --- |
| *1* OR = Odds Ratio, CI = Confidence Interval | | | | | | | | | |

### **4. Categorical fragmentation and Continuous neighbours (in MS)**

| **Characteristic** | **OR**1 | **95% CI**1 | **p-value** |
| --- | --- | --- | --- |
| CountNeighbourbTB | 1.45 | 1.40, 1.51 | <0.001 |
| fragment\_category |  |  |  |
| Not\_fragmented | — | — |  |
| Little\_fragmentation | 0.83 | 0.72, 0.96 | 0.014 |
| Medium\_fragmentation | 0.96 | 0.81, 1.12 | 0.6 |
| High\_fragmentation | 1.06 | 0.87, 1.30 | 0.6 |
| Very\_High\_fragmentation | 0.73 | 0.59, 0.91 | 0.004 |
|  |  |  |  |
| --- | --- | --- | --- |
| *1* OR = Odds Ratio, CI = Confidence Interval | | | |

| **Characteristic** | **OR**1 | **95% CI**1 | **p-value** |
| --- | --- | --- | --- |
| CountNeighbourbTB | 1.44 | 1.25, 1.65 | <0.001 |
| fragment\_category |  |  |  |
| Not\_fragmented | — | — |  |
| Little\_fragmentation | 0.78 | 0.66, 0.94 | 0.008 |
| Medium\_fragmentation | 0.97 | 0.79, 1.18 | 0.7 |
| High\_fragmentation | 1.17 | 0.90, 1.52 | 0.2 |
| Very\_High\_fragmentation | 0.80 | 0.59, 1.07 | 0.13 |
| CountNeighbourbTB \* fragment\_category |  |  |  |
| CountNeighbourbTB \* Little\_fragmentation | 1.07 | 0.92, 1.24 | 0.4 |
| CountNeighbourbTB \* Medium\_fragmentation | 1.00 | 0.86, 1.16 | >0.9 |
| CountNeighbourbTB \* High\_fragmentation | 0.95 | 0.81, 1.12 | 0.5 |
| CountNeighbourbTB \* Very\_High\_fragmentation | 0.97 | 0.83, 1.14 | 0.7 |
|  |  |  |  |
| --- | --- | --- | --- |
| *1* OR = Odds Ratio, CI = Confidence Interval | | | |

The univariable model is improved by the addition of bTB positive neighbours


|  | loglik | Chisq | Df | P(>|Chi|) |
| --- | --- | --- | --- | --- |
| 1 | -3187.45 |  |  |  |
| 2 | -2909.70 | 555.50 | 1 | 0.0000 |

No evidence that an interaction term is needed

```
## <!-- html table generated in R 4.1.0 by xtable 1.8-4 package -->
## <!-- Tue Sep 14 11:51:06 2021 -->
## <table border=1>
## <tr> <th>  </th> <th> loglik </th> <th> Chisq </th> <th> Df </th> <th> P(&gt;|Chi|) </th>  </tr>
##   <tr> <td> 1 </td> <td align="right"> -2909.70 </td> <td align="right">  </td> <td align="right">  </td> <td align="right">  </td> </tr>
##   <tr> <td> 2 </td> <td align="right"> -2906.31 </td> <td align="right"> 6.80 </td> <td align="right"> 4 </td> <td align="right"> 0.1471 </td> </tr>
##    </table>
```

| **Characteristic** | Unadjusted Analysis | | | Adjusted Analysis | | | Adjusted Analysis (Interaction | | |
| --- | --- | --- | --- | --- | --- | --- | --- | --- | --- |
|  | **OR**1 | **95% CI**1 | **p-value** | **OR**1 | **95% CI**1 | **p-value** | **OR**1 | **95% CI**1 | **p-value** |
| fragment\_category |  |  |  |  |  |  |  |  |  |
| Not\_fragmented | — | — |  | — | — |  | — | — |  |
| Little\_fragmentation | 0.97 | 0.84, 1.11 | 0.7 | 0.83 | 0.72, 0.96 | 0.014 | 0.78 | 0.66, 0.94 | 0.008 |
| Medium\_fragmentation | 1.25 | 1.07, 1.46 | 0.004 | 0.96 | 0.81, 1.12 | 0.6 | 0.97 | 0.79, 1.18 | 0.7 |
| High\_fragmentation | 1.56 | 1.30, 1.88 | <0.001 | 1.06 | 0.87, 1.30 | 0.6 | 1.17 | 0.90, 1.52 | 0.2 |
| Very\_High\_fragmentation | 1.36 | 1.12, 1.65 | 0.002 | 0.73 | 0.59, 0.91 | 0.004 | 0.80 | 0.59, 1.07 | 0.13 |
| CountNeighbourbTB | 1.45 | 1.40, 1.50 | <0.001 | 1.45 | 1.40, 1.51 | <0.001 | 1.44 | 1.25, 1.65 | <0.001 |
| CountNeighbourbTB \* fragment\_category |  |  |  |  |  |  |  |  |  |
| CountNeighbourbTB \* Little\_fragmentation |  |  |  |  |  |  | 1.07 | 0.92, 1.24 | 0.4 |
| CountNeighbourbTB \* Medium\_fragmentation |  |  |  |  |  |  | 1.00 | 0.86, 1.16 | >0.9 |
| CountNeighbourbTB \* High\_fragmentation |  |  |  |  |  |  | 0.95 | 0.81, 1.12 | 0.5 |
| CountNeighbourbTB \* Very\_High\_fragmentation |  |  |  |  |  |  | 0.97 | 0.83, 1.14 | 0.7 |
|  |  |  |  |  |  |  |  |  |  |
| --- | --- | --- | --- | --- | --- | --- | --- | --- | --- |
| *1* OR = Odds Ratio, CI = Confidence Interval | | | | | | | | | |

Confounding in fragmentation - looks like the presence of bTB positive neighbours confounds the relationship with fragmentation and breakdown risk.

```
## [1] "14% difference between non adjusted and adjusted coefficient"
## [2] "24% difference between non adjusted and adjusted coefficient"
## [3] "32% difference between non adjusted and adjusted coefficient"
## [4] "46% difference between non adjusted and adjusted coefficient"
```

```
## [1] "1% difference between non adjusted and adjusted coefficient"
```

Explore confounding more - what's going on?

Larger farms more are likely to have bTB positive neighbours. The table below shows that 75% of larger farms have at least 1 bTB positive neighbour, compared to only 37% of smaller farms

```
## # A tibble: 5 x 5
##   fragment_category          Q1   med    Q3   max
##   <fct>                   <dbl> <dbl> <dbl> <dbl>
## 1 Not_fragmented              0     0     1     5
## 2 Little_fragmentation        0     1     2    11
## 3 Medium_fragmentation        0     1     2    14
## 4 High_fragmentation          1     2     3    13
## 5 Very_High_fragmentation     1     2     4    14
```

```
Fig4Frag
```

| **Variable** | **Not\_fragmented**, N = 1,0141 | **Little\_fragmentation**, N = 4,2001 | **Medium\_fragmentation**, N = 2,2271 | **High\_fragmentation**, N = 9661 | **Very\_High\_fragmentation**, N = 8671 |
| --- | --- | --- | --- | --- | --- |
| NeighbourbTB | 461 (45%) | 2,298 (55%) | 1,531 (69%) | 725 (75%) | 732 (84%) |
|  |  |  |  |  |  |
| --- | --- | --- | --- | --- | --- |
| *1* n (%) | | | | | |

### 4B FRAGMENT DISPERSAL

### 1. COntinuous dispersal and binary neighbours (not in MS)

| **Characteristic** | **OR**1 | **95% CI**1 | **p-value** |
| --- | --- | --- | --- |
| NeighbourbTB | 2.20 | 2.01, 2.41 | <0.001 |
| median\_distance\_fragments\_km\_scaled | 1.00 | 1.00, 1.00 | 0.7 |
|  |  |  |  |
| --- | --- | --- | --- |
| *1* OR = Odds Ratio, CI = Confidence Interval | | | |

| **Characteristic** | **OR**1 | **95% CI**1 | **p-value** |
| --- | --- | --- | --- |
| NeighbourbTB | 2.35 | 2.11, 2.61 | <0.001 |
| median\_distance\_fragments\_km\_scaled | 1.00 | 1.00, 1.00 | 0.031 |
| NeighbourbTB \* median\_distance\_fragments\_km\_scaled | 1.00 | 1.00, 1.00 | 0.018 |
|  |  |  |  |
| --- | --- | --- | --- |
| *1* OR = Odds Ratio, CI = Confidence Interval | | | |

| **Characteristic** | Unadjusted Analysis | | | Adjusted Analysis | | | Adjusted Analysis (Interaction) | | |
| --- | --- | --- | --- | --- | --- | --- | --- | --- | --- |
|  | **OR**1 | **95% CI**1 | **p-value** | **OR**1 | **95% CI**1 | **p-value** | **OR**1 | **95% CI**1 | **p-value** |
| median\_distance\_fragments\_km\_scaled | 1.00 | 1.00, 1.00 | 0.030 | 1.00 | 1.00, 1.00 | 0.7 | 1.00 | 1.00, 1.00 | 0.031 |
| NeighbourbTB | 2.20 | 2.01, 2.41 | <0.001 | 2.20 | 2.01, 2.41 | <0.001 | 2.35 | 2.11, 2.61 | <0.001 |
| NeighbourbTB \* median\_distance\_fragments\_km\_scaled |  |  |  |  |  |  | 1.00 | 1.00, 1.00 | 0.018 |
|  |  |  |  |  |  |  |  |  |  |
| --- | --- | --- | --- | --- | --- | --- | --- | --- | --- |
| *1* OR = Odds Ratio, CI = Confidence Interval | | | | | | | | | |

### 2. Continuous dispersal and continuous neighbours (not in MS)

| **Characteristic** | **OR**1 | **95% CI**1 | **p-value** |
| --- | --- | --- | --- |
| CountNeighbourbTB | 1.45 | 1.40, 1.50 | <0.001 |
| median\_distance\_fragments\_km\_scaled | 1.00 | 1.00, 1.00 | 0.5 |
|  |  |  |  |
| --- | --- | --- | --- |
| *1* OR = Odds Ratio, CI = Confidence Interval | | | |

| **Characteristic** | **OR**1 | **95% CI**1 | **p-value** |
| --- | --- | --- | --- |
| CountNeighbourbTB | 1.54 | 1.47, 1.60 | <0.001 |
| median\_distance\_fragments\_km\_scaled | 1.00 | 1.00, 1.00 | 0.007 |
| CountNeighbourbTB \* median\_distance\_fragments\_km\_scaled | 1.00 | 1.00, 1.00 | <0.001 |
|  |  |  |  |
| --- | --- | --- | --- |
| *1* OR = Odds Ratio, CI = Confidence Interval | | | |

| **Characteristic** | Unadjusted Analysis | | | Adjusted Analysis | | | Adjusted Analysis (Interaction) | | |
| --- | --- | --- | --- | --- | --- | --- | --- | --- | --- |
|  | **OR**1 | **95% CI**1 | **p-value** | **OR**1 | **95% CI**1 | **p-value** | **OR**1 | **95% CI**1 | **p-value** |
| median\_distance\_fragments\_km\_scaled | 1.00 | 1.00, 1.00 | 0.030 | 1.00 | 1.00, 1.00 | 0.5 | 1.00 | 1.00, 1.00 | 0.007 |
| CountNeighbourbTB | 1.45 | 1.40, 1.50 | <0.001 | 1.45 | 1.40, 1.50 | <0.001 | 1.54 | 1.47, 1.60 | <0.001 |
| CountNeighbourbTB \* median\_distance\_fragments\_km\_scaled |  |  |  |  |  |  | 1.00 | 1.00, 1.00 | <0.001 |
|  |  |  |  |  |  |  |  |  |  |
| --- | --- | --- | --- | --- | --- | --- | --- | --- | --- |
| *1* OR = Odds Ratio, CI = Confidence Interval | | | | | | | | | |

### 3. Binary dispersal and binary neighbours (not in MS)

| **Characteristic** | **OR**1 | **95% CI**1 | **p-value** |
| --- | --- | --- | --- |
| NeighbourbTB | 2.14 | 1.95, 2.34 | <0.001 |
| HighVeryHighDisp |  |  |  |
| SmallMedium | — | — |  |
| HighVeryHigh | 1.23 | 1.12, 1.35 | <0.001 |
|  |  |  |  |
| --- | --- | --- | --- |
| *1* OR = Odds Ratio, CI = Confidence Interval | | | |

| **Characteristic** | **OR**1 | **95% CI**1 | **p-value** |
| --- | --- | --- | --- |
| NeighbourbTB | 2.18 | 1.91, 2.49 | <0.001 |
| HighVeryHighDisp |  |  |  |
| SmallMedium | — | — |  |
| HighVeryHigh | 1.26 | 1.09, 1.45 | 0.001 |
| NeighbourbTB \* HighVeryHighDisp |  |  |  |
| NeighbourbTB \* HighVeryHigh | 0.96 | 0.81, 1.15 | 0.7 |
|  |  |  |  |
| --- | --- | --- | --- |
| *1* OR = Odds Ratio, CI = Confidence Interval | | | |

| **Characteristic** | Unadjusted Analysis | | | Adjusted Analysis | | | Adjusted Analysis (Interaction) | | |
| --- | --- | --- | --- | --- | --- | --- | --- | --- | --- |
|  | **OR**1 | **95% CI**1 | **p-value** | **OR**1 | **95% CI**1 | **p-value** | **OR**1 | **95% CI**1 | **p-value** |
| HighVeryHighDisp |  |  |  |  |  |  |  |  |  |
| SmallMedium | — | — |  | — | — |  | — | — |  |
| HighVeryHigh | 1.37 | 1.25, 1.49 | <0.001 | 1.23 | 1.12, 1.35 | <0.001 | 1.26 | 1.09, 1.45 | 0.001 |
| NeighbourbTB | 2.20 | 2.01, 2.41 | <0.001 | 2.14 | 1.95, 2.34 | <0.001 | 2.18 | 1.91, 2.49 | <0.001 |
| NeighbourbTB \* HighVeryHighDisp |  |  |  |  |  |  |  |  |  |
| NeighbourbTB \* HighVeryHigh |  |  |  |  |  |  | 0.96 | 0.81, 1.15 | 0.7 |
|  |  |  |  |  |  |  |  |  |  |
| --- | --- | --- | --- | --- | --- | --- | --- | --- | --- |
| *1* OR = Odds Ratio, CI = Confidence Interval | | | | | | | | | |

### **4. Binary dispersal and continuous neighbours (in MS)**

| **Characteristic** | **OR**1 | **95% CI**1 | **p-value** |
| --- | --- | --- | --- |
| CountNeighbourbTB | 1.44 | 1.39, 1.49 | <0.001 |
| fragment\_distance\_category |  |  |  |
| Low | — | — |  |
| Medium | 0.84 | 0.73, 0.97 | 0.016 |
| High | 1.01 | 0.87, 1.17 | 0.9 |
| Very High | 1.01 | 0.88, 1.16 | 0.9 |
|  |  |  |  |
| --- | --- | --- | --- |
| *1* OR = Odds Ratio, CI = Confidence Interval | | | |

| **Characteristic** | **OR**1 | **95% CI**1 | **p-value** |
| --- | --- | --- | --- |
| CountNeighbourbTB | 1.63 | 1.44, 1.84 | <0.001 |
| fragment\_distance\_category |  |  |  |
| Low | — | — |  |
| Medium | 0.83 | 0.70, 0.99 | 0.037 |
| High | 1.09 | 0.91, 1.30 | 0.4 |
| Very High | 1.19 | 1.00, 1.41 | 0.053 |
| CountNeighbourbTB \* fragment\_distance\_category |  |  |  |
| CountNeighbourbTB \* Medium | 0.97 | 0.84, 1.12 | 0.7 |
| CountNeighbourbTB \* High | 0.88 | 0.77, 1.01 | 0.068 |
| CountNeighbourbTB \* Very High | 0.83 | 0.73, 0.95 | 0.007 |
|  |  |  |  |
| --- | --- | --- | --- |
| *1* OR = Odds Ratio, CI = Confidence Interval | | | |

| **Characteristic** | Unadjusted Analysis | | | Adjusted Analysis | | | Adjusted Analysis (Interaction) | | |
| --- | --- | --- | --- | --- | --- | --- | --- | --- | --- |
|  | **OR**1 | **95% CI**1 | **p-value** | **OR**1 | **95% CI**1 | **p-value** | **OR**1 | **95% CI**1 | **p-value** |
| fragment\_distance\_category |  |  |  |  |  |  |  |  |  |
| Low | — | — |  | — | — |  | — | — |  |
| Medium | 0.97 | 0.85, 1.11 | 0.6 | 0.84 | 0.73, 0.97 | 0.016 | 0.83 | 0.70, 0.99 | 0.037 |
| High | 1.33 | 1.16, 1.52 | <0.001 | 1.01 | 0.87, 1.17 | 0.9 | 1.09 | 0.91, 1.30 | 0.4 |
| Very High | 1.35 | 1.19, 1.55 | <0.001 | 1.01 | 0.88, 1.16 | 0.9 | 1.19 | 1.00, 1.41 | 0.053 |
| CountNeighbourbTB | 1.45 | 1.40, 1.50 | <0.001 | 1.44 | 1.39, 1.49 | <0.001 | 1.63 | 1.44, 1.84 | <0.001 |
| CountNeighbourbTB \* fragment\_distance\_category |  |  |  |  |  |  |  |  |  |
| CountNeighbourbTB \* Medium |  |  |  |  |  |  | 0.97 | 0.84, 1.12 | 0.7 |
| CountNeighbourbTB \* High |  |  |  |  |  |  | 0.88 | 0.77, 1.01 | 0.068 |
| CountNeighbourbTB \* Very High |  |  |  |  |  |  | 0.83 | 0.73, 0.95 | 0.007 |
|  |  |  |  |  |  |  |  |  |  |
| --- | --- | --- | --- | --- | --- | --- | --- | --- | --- |
| *1* OR = Odds Ratio, CI = Confidence Interval | | | | | | | | | |

The univariable model is improved by the addition of bTB positive neighbours


|  | loglik | Chisq | Df | P(>|Chi|) |
| --- | --- | --- | --- | --- |
| 1 | -3188.68 |  |  |  |
| 2 | -2914.72 | 547.92 | 1 | 0.0000 |

An interaction term is needed:


|  | loglik | Chisq | Df | P(>|Chi|) |
| --- | --- | --- | --- | --- |
| 1 | -2914.72 |  |  |  |
| 2 | -2907.27 | 14.91 | 3 | 0.0019 |

```
## [1] "13% difference between non adjusted and adjusted coefficient"
## [2] "24% difference between non adjusted and adjusted coefficient"
## [3] "25% difference between non adjusted and adjusted coefficient"
```

```
## [1] "0% difference between non adjusted and adjusted coefficient"
```

Investigate relationships

```
## # A tibble: 4 x 5
##   fragment_distance_category    Q1   med    Q3   max
##   <fct>                      <dbl> <dbl> <dbl> <dbl>
## 1 Low                            0     0     1     6
## 2 Medium                         0     1     2    10
## 3 High                           0     1     2    13
## 4 Very High                      0     1     2    14
```

```
Fig4Disp
```

| **Variable** | **Low**, N = 1,4951 | **Medium**, N = 2,4541 | **High**, N = 2,7071 | **Very High**, N = 2,6181 |
| --- | --- | --- | --- | --- |
| NeighbourbTB | 626 (42%) | 1,411 (57%) | 1,918 (71%) | 1,792 (68%) |
|  |  |  |  |  |
| --- | --- | --- | --- | --- |
| *1* n (%) | | | | |

### 4C CONTACT METRICS

### 1. COntinuous contact and binary neighbours (not in MS)

| **Characteristic** | **OR**1 | **95% CI**1 | **p-value** |
| --- | --- | --- | --- |
| NeighbourbTB | 2.06 | 1.88, 2.27 | <0.001 |
| total\_shared\_boundary\_grazing\_km | 1.04 | 1.03, 1.06 | <0.001 |
|  |  |  |  |
| --- | --- | --- | --- |
| *1* OR = Odds Ratio, CI = Confidence Interval | | | |

| **Characteristic** | **OR**1 | **95% CI**1 | **p-value** |
| --- | --- | --- | --- |
| NeighbourbTB | 2.31 | 1.99, 2.68 | <0.001 |
| total\_shared\_boundary\_grazing\_km | 1.07 | 1.04, 1.10 | <0.001 |
| NeighbourbTB \* total\_shared\_boundary\_grazing\_km | 0.97 | 0.94, 1.00 | 0.050 |
|  |  |  |  |
| --- | --- | --- | --- |
| *1* OR = Odds Ratio, CI = Confidence Interval | | | |

| **Characteristic** | Unadjusted Analysis | | | Adjusted Analysis | | | Adjusted Analysis (Interaction) | | |
| --- | --- | --- | --- | --- | --- | --- | --- | --- | --- |
|  | **OR**1 | **95% CI**1 | **p-value** | **OR**1 | **95% CI**1 | **p-value** | **OR**1 | **95% CI**1 | **p-value** |
| total\_shared\_boundary\_grazing\_km | 1.07 | 1.06, 1.09 | <0.001 | 1.04 | 1.03, 1.06 | <0.001 | 1.07 | 1.04, 1.10 | <0.001 |
| NeighbourbTB | 2.20 | 2.01, 2.41 | <0.001 | 2.06 | 1.88, 2.27 | <0.001 | 2.31 | 1.99, 2.68 | <0.001 |
| NeighbourbTB \* total\_shared\_boundary\_grazing\_km |  |  |  |  |  |  | 0.97 | 0.94, 1.00 | 0.050 |
|  |  |  |  |  |  |  |  |  |  |
| --- | --- | --- | --- | --- | --- | --- | --- | --- | --- |
| *1* OR = Odds Ratio, CI = Confidence Interval | | | | | | | | | |

### 2. Continuous contact and continuous neighbours (not in MS)

| **Characteristic** | **OR**1 | **95% CI**1 | **p-value** |
| --- | --- | --- | --- |
| CountNeighbourbTB | 1.44 | 1.39, 1.50 | <0.001 |
| total\_shared\_boundary\_grazing\_km | 1.00 | 0.99, 1.02 | 0.8 |
|  |  |  |  |
| --- | --- | --- | --- |
| *1* OR = Odds Ratio, CI = Confidence Interval | | | |

| **Characteristic** | **OR**1 | **95% CI**1 | **p-value** |
| --- | --- | --- | --- |
| CountNeighbourbTB | 1.58 | 1.50, 1.66 | <0.001 |
| total\_shared\_boundary\_grazing\_km | 1.03 | 1.01, 1.05 | 0.003 |
| CountNeighbourbTB \* total\_shared\_boundary\_grazing\_km | 0.99 | 0.98, 0.99 | <0.001 |
|  |  |  |  |
| --- | --- | --- | --- |
| *1* OR = Odds Ratio, CI = Confidence Interval | | | |

| **Characteristic** | Unadjusted Analysis | | | Adjusted Analysis | | | Adjusted Analysis (Interaction) | | |
| --- | --- | --- | --- | --- | --- | --- | --- | --- | --- |
|  | **OR**1 | **95% CI**1 | **p-value** | **OR**1 | **95% CI**1 | **p-value** | **OR**1 | **95% CI**1 | **p-value** |
| total\_shared\_boundary\_grazing\_km | 1.07 | 1.06, 1.09 | <0.001 | 1.00 | 0.99, 1.02 | 0.8 | 1.03 | 1.01, 1.05 | 0.003 |
| CountNeighbourbTB | 1.45 | 1.40, 1.50 | <0.001 | 1.44 | 1.39, 1.50 | <0.001 | 1.58 | 1.50, 1.66 | <0.001 |
| CountNeighbourbTB \* total\_shared\_boundary\_grazing\_km |  |  |  |  |  |  | 0.99 | 0.98, 0.99 | <0.001 |
|  |  |  |  |  |  |  |  |  |  |
| --- | --- | --- | --- | --- | --- | --- | --- | --- | --- |
| *1* OR = Odds Ratio, CI = Confidence Interval | | | | | | | | | |

### 3. Categorical contact & binary neighbours (not in MS)

| **Characteristic** | **OR**1 | **95% CI**1 | **p-value** |
| --- | --- | --- | --- |
| NeighbourbTB | 2.10 | 1.91, 2.31 | <0.001 |
| neighbour\_contact\_category |  |  |  |
| Low | — | — |  |
| Medium | 0.92 | 0.80, 1.06 | 0.3 |
| High | 0.97 | 0.84, 1.12 | 0.7 |
| Very High | 1.25 | 1.08, 1.46 | 0.003 |
|  |  |  |  |
| --- | --- | --- | --- |
| *1* OR = Odds Ratio, CI = Confidence Interval | | | |

| **Characteristic** | **OR**1 | **95% CI**1 | **p-value** |
| --- | --- | --- | --- |
| NeighbourbTB | 2.34 | 1.86, 2.93 | <0.001 |
| neighbour\_contact\_category |  |  |  |
| Low | — | — |  |
| Medium | 0.89 | 0.74, 1.07 | 0.2 |
| High | 1.11 | 0.91, 1.35 | 0.3 |
| Very High | 1.37 | 1.10, 1.71 | 0.005 |
| NeighbourbTB \* neighbour\_contact\_category |  |  |  |
| NeighbourbTB \* Medium | 1.04 | 0.78, 1.39 | 0.8 |
| NeighbourbTB \* High | 0.78 | 0.59, 1.04 | 0.090 |
| NeighbourbTB \* Very High | 0.84 | 0.64, 1.12 | 0.2 |
|  |  |  |  |
| --- | --- | --- | --- |
| *1* OR = Odds Ratio, CI = Confidence Interval | | | |

| **Characteristic** | Unadjusted Analysis | | | Adjusted Analysis | | | Adjusted Analysis (Interaction) | | |
| --- | --- | --- | --- | --- | --- | --- | --- | --- | --- |
|  | **OR**1 | **95% CI**1 | **p-value** | **OR**1 | **95% CI**1 | **p-value** | **OR**1 | **95% CI**1 | **p-value** |
| neighbour\_contact\_category |  |  |  |  |  |  |  |  |  |
| Low | — | — |  | — | — |  | — | — |  |
| Medium | 1.02 | 0.89, 1.18 | 0.7 | 0.92 | 0.80, 1.06 | 0.3 | 0.89 | 0.74, 1.07 | 0.2 |
| High | 1.20 | 1.04, 1.38 | 0.011 | 0.97 | 0.84, 1.12 | 0.7 | 1.11 | 0.91, 1.35 | 0.3 |
| Very High | 1.72 | 1.49, 1.98 | <0.001 | 1.25 | 1.08, 1.46 | 0.003 | 1.37 | 1.10, 1.71 | 0.005 |
| NeighbourbTB | 2.20 | 2.01, 2.41 | <0.001 | 2.10 | 1.91, 2.31 | <0.001 | 2.34 | 1.86, 2.93 | <0.001 |
| NeighbourbTB \* neighbour\_contact\_category |  |  |  |  |  |  |  |  |  |
| NeighbourbTB \* Medium |  |  |  |  |  |  | 1.04 | 0.78, 1.39 | 0.8 |
| NeighbourbTB \* High |  |  |  |  |  |  | 0.78 | 0.59, 1.04 | 0.090 |
| NeighbourbTB \* Very High |  |  |  |  |  |  | 0.84 | 0.64, 1.12 | 0.2 |
|  |  |  |  |  |  |  |  |  |  |
| --- | --- | --- | --- | --- | --- | --- | --- | --- | --- |
| *1* OR = Odds Ratio, CI = Confidence Interval | | | | | | | | | |

### **4. Categorical contact & continuous neighbours (in MS)**

| **Characteristic** | **OR**1 | **95% CI**1 | **p-value** |
| --- | --- | --- | --- |
| CountNeighbourbTB | 1.44 | 1.39, 1.49 | <0.001 |
| neighbour\_contact\_category |  |  |  |
| Low | — | — |  |
| Medium | 0.91 | 0.79, 1.05 | 0.2 |
| High | 0.95 | 0.82, 1.10 | 0.5 |
| Very High | 1.02 | 0.87, 1.19 | 0.8 |
|  |  |  |  |
| --- | --- | --- | --- |
| *1* OR = Odds Ratio, CI = Confidence Interval | | | |

| **Characteristic** | **OR**1 | **95% CI**1 | **p-value** |
| --- | --- | --- | --- |
| CountNeighbourbTB | 1.46 | 1.29, 1.66 | <0.001 |
| neighbour\_contact\_category |  |  |  |
| Low | — | — |  |
| Medium | 0.79 | 0.66, 0.94 | 0.009 |
| High | 0.85 | 0.71, 1.02 | 0.074 |
| Very High | 1.22 | 1.01, 1.46 | 0.035 |
| CountNeighbourbTB \* neighbour\_contact\_category |  |  |  |
| CountNeighbourbTB \* Medium | 1.21 | 1.03, 1.42 | 0.023 |
| CountNeighbourbTB \* High | 1.11 | 0.96, 1.29 | 0.2 |
| CountNeighbourbTB \* Very High | 0.90 | 0.79, 1.03 | 0.12 |
|  |  |  |  |
| --- | --- | --- | --- |
| *1* OR = Odds Ratio, CI = Confidence Interval | | | |

| **Characteristic** | Unadjusted Analysis | | | Adjusted Analysis | | | Adjusted Analysis (Interaction) | | |
| --- | --- | --- | --- | --- | --- | --- | --- | --- | --- |
|  | **OR**1 | **95% CI**1 | **p-value** | **OR**1 | **95% CI**1 | **p-value** | **OR**1 | **95% CI**1 | **p-value** |
| neighbour\_contact\_category |  |  |  |  |  |  |  |  |  |
| Low | — | — |  | — | — |  | — | — |  |
| Medium | 1.02 | 0.89, 1.18 | 0.7 | 0.91 | 0.79, 1.05 | 0.2 | 0.79 | 0.66, 0.94 | 0.009 |
| High | 1.20 | 1.04, 1.38 | 0.011 | 0.95 | 0.82, 1.10 | 0.5 | 0.85 | 0.71, 1.02 | 0.074 |
| Very High | 1.72 | 1.49, 1.98 | <0.001 | 1.02 | 0.87, 1.19 | 0.8 | 1.22 | 1.01, 1.46 | 0.035 |
| CountNeighbourbTB | 1.45 | 1.40, 1.50 | <0.001 | 1.44 | 1.39, 1.49 | <0.001 | 1.46 | 1.29, 1.66 | <0.001 |
| CountNeighbourbTB \* neighbour\_contact\_category |  |  |  |  |  |  |  |  |  |
| CountNeighbourbTB \* Medium |  |  |  |  |  |  | 1.21 | 1.03, 1.42 | 0.023 |
| CountNeighbourbTB \* High |  |  |  |  |  |  | 1.11 | 0.96, 1.29 | 0.2 |
| CountNeighbourbTB \* Very High |  |  |  |  |  |  | 0.90 | 0.79, 1.03 | 0.12 |
|  |  |  |  |  |  |  |  |  |  |
| --- | --- | --- | --- | --- | --- | --- | --- | --- | --- |
| *1* OR = Odds Ratio, CI = Confidence Interval | | | | | | | | | |

The univariable model is improved by the addition of bTB positive neighbours


|  | loglik | Chisq | Df | P(>|Chi|) |
| --- | --- | --- | --- | --- |
| 1 | -3169.94 |  |  |  |
| 2 | -2919.27 | 501.35 | 1 | 0.0000 |

No evidence that an interaction term is needed


|  | loglik | Chisq | Df | P(>|Chi|) |
| --- | --- | --- | --- | --- |
| 1 | -2919.27 |  |  |  |
| 2 | -2897.13 | 44.27 | 3 | 0.0000 |

coefficient changes - 10% rule for contact metrics and neighbour bTB status

```
## [1] "11% difference between non adjusted and adjusted coefficient"
## [2] "20% difference between non adjusted and adjusted coefficient"
## [3] "41% difference between non adjusted and adjusted coefficient"
```

```
## [1] "0.53% difference between non adjusted and adjusted coefficient"
```

Explore confounding more - what's going on?

```
## # A tibble: 4 x 5
##   neighbour_contact_category    Q1   med    Q3   max
##   <fct>                      <dbl> <dbl> <dbl> <dbl>
## 1 Low                            0     0     1     6
## 2 Medium                         0     0     1     8
## 3 High                           0     1     2     8
## 4 Very High                      1     2     3    14
```

```
Fig4Contact
```

| **Variable** | **Low**, N = 1,4071 | **Medium**, N = 2,0041 | **High**, N = 2,4361 | **Very High**, N = 3,4271 |
| --- | --- | --- | --- | --- |
| NeighbourbTB | 503 (36%) | 991 (49%) | 1,548 (64%) | 2,705 (79%) |
|  |  |  |  |  |
| --- | --- | --- | --- | --- |
| *1* n (%) | | | | |
